# Kinetically Controlled and Nonequilibrium Assembly of Block Copolymers in Solution

**DOI:** 10.1021/jacs.4c03314

**Published:** 2024-07-05

**Authors:** Stephen D. P. Fielden

**Affiliations:** School of Chemistry, University of Birmingham, Edgbaston, Birmingham B15 2TT, United Kingdom

## Abstract

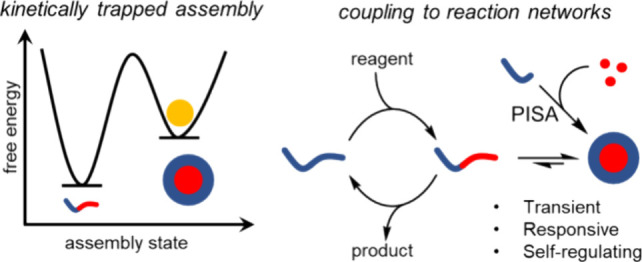

Covalent polymers are versatile macromolecules that have found widespread use in society. Contemporary methods of polymerization have made it possible to construct sequence polymers, including block copolymers, with high precision. Such copolymers assemble in solution when the blocks have differing solubilities. This produces nano- and microparticles of various shapes and sizes. While it is straightforward to draw an analogy between such amphiphilic block copolymers and phospholipids, these two classes of molecules show quite different assembly characteristics. In particular, block copolymers often assemble under kinetic control, thus producing nonequilibrium structures. This leads to a rich variety of behaviors being observed in block copolymer assembly, such as pathway dependence (e.g., thermal history), nonergodicity and responsiveness. The dynamics of polymer assemblies can be readily controlled using changes in environmental conditions and/or integrating functional groups situated on polymers with external chemical reactions. This perspective highlights that kinetic control is both pervasive and a useful attribute in the mechanics of block copolymer assembly. Recent examples are highlighted in order to show that toggling between static and dynamic behavior can be used to generate, manipulate and dismantle nonequilibrium states. New methods to control the kinetics of block copolymer assembly will provide endless unanticipated applications in materials science, biomimicry and medicine.

## Introduction

The discovery and application of synthetic polymers have revolutionized modern society. They have reduced our reliance on macroscopic materials derived from biological products such as wood and leather. Using polymers to develop new nanotechnology will cause a similar revolution in the coming decades.^1^ Currently, biological-derived assemblies (made from phospholipids) are used much more frequently than polymers for exploring compartmentalization in solution at the nanoscale.^2^ While phospholipid assemblies are perfect for studying biological systems in water, they offer limited structural diversity, resulting in a narrow range of assembly architectures and conditions. Block copolymers can theoretically assemble in any solution and thus offer a much larger range of applicability. This is because the composition, topology and length of polymer chains are readily controlled using synthetic chemistry.^3^

**Figure 1:**
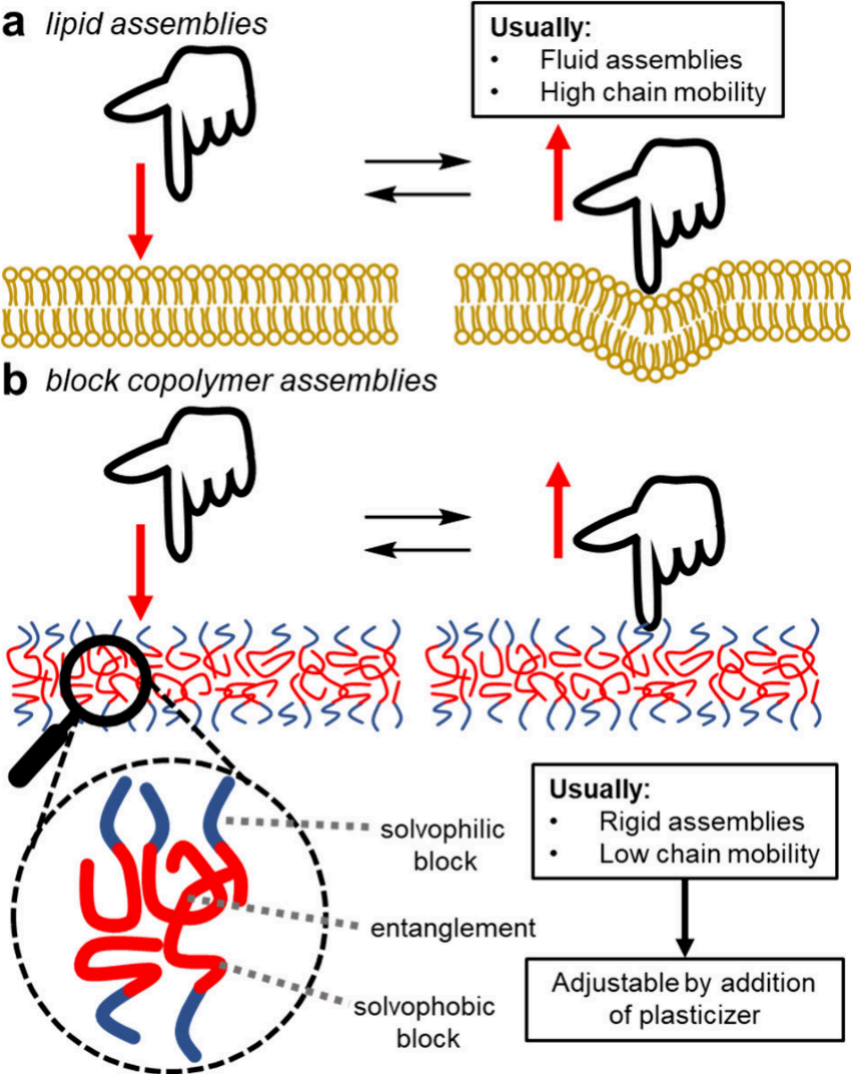
Comparison between (a) phospholipid assemblies, which generally possess fluid-like behavior and (b) block copolymer assemblies, which are generally rigid. These assemblies are harder to deform due to their glassy nature and physical cross-linking arising from chain entanglement.

Another important distinction between synthetic polymers and phospholipids is chain length. Phospholipids are formed of fatty acids with carbon chains that are normally around 20 atoms long. Contrastingly, polymer chains can be formed of hundreds of monomer units and thus are much longer. This has several important ramifications for polymer chain dynamics: (1) A linear poly(ethylene) chain needs to be around 250 carbon atoms long to entangle.^4,5^ Unlike phospholipids, some polymer chains are able to entangle, leading to transient physical cross-links. This means that interchain interactions are profoundly different for phospholipids and polymers.^6^ (2) With systems at or near equilibrium, surfactant chains exchange between assemblies via stepwise expulsion/insertion of individual chains (known as the Aniansson-Wall mechanism).^7−9^ This is because the activation energy of chain expulsion is lower than for processes involving entire assemblies, such as fission or fusion (see later for exceptions to this with nonequilibrium systems). The activation energy for ejection of a surfactant increases with solvophobic block chain length, due to the requirement to expose a greater surface area of insoluble polymer to the solvent.^10^ Thus, exchange kinetics for polymer assemblies can be several orders of magnitude slower than for phospholipids.^11^ (3) Under ambient conditions, phospholipids are usually molten and polymers are often glassy. This means that chain mobility is rapid within assembled phospholipids, leading to fluid-like behavior. Conversely, mobility of frozen polymer chains within the solvophobic core of assemblies is often very slow or completely suppressed (Figure 1).^12^

The lack of chain mobility within polymer assemblies means that nonequilibrium (or metastable) structures can persist; the barrier to structural rearrangement is significantly greater than the available thermal energy.^13^ Ultimately, this means polymer assembly is often subject to kinetic control. This leads to a rich variety of *static* and *dynamic* behaviors and properties found in polymer assemblies. These are not observed in phospholipid assemblies without the presence of additional biological machinery. Understanding how the kinetics of polymer assembly can be manipulated is still a relatively young field, but offers much potential in the development of adaptive and responsive (“smart”) nanotechnology. Here I highlight several examples of kinetic control used to produce both static and dynamic polymer assemblies, and suggest how this could be exploited in the future. Specific focus will be given to assemblies of block copolymers in solution.

## Kinetically Controlled Assembly of Preformed Block Copolymers

**Figure 2:**
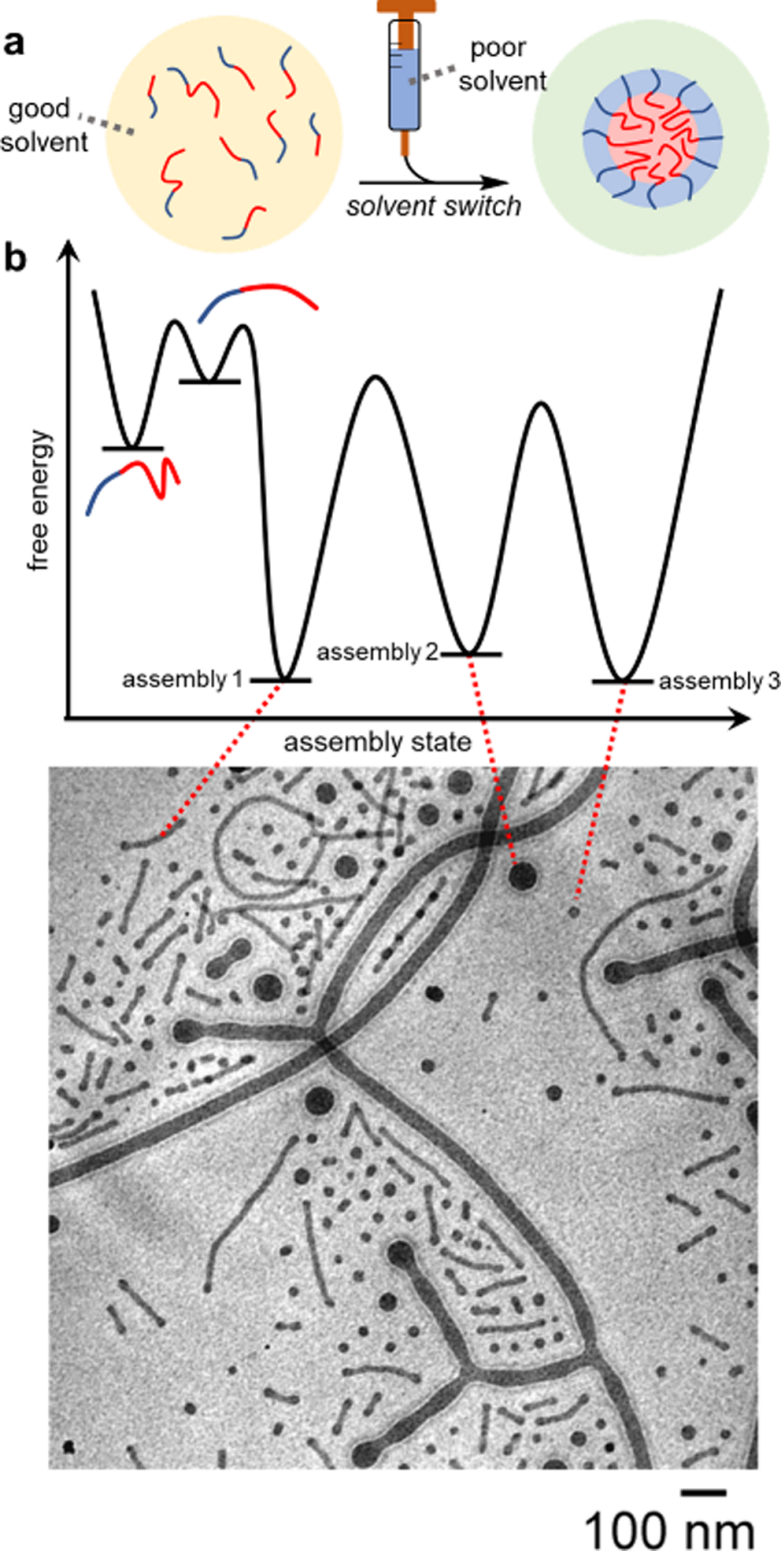
(a) Cartoon depicting a solvent switch procedure. Slow addition of the poor solvent gives the polymer chains a greater chance of assembling into a thermodynamically favored state. (b) Qualitative energy diagram to illustrate the different states of a diblock copolymer in a solvent system that favors assembly. The polymer chain has higher free energy when dissolved in solution than when assembled into a structure that decreases exposure of the solvophobic block to the solvent. Extension of the solvophobic block in solution is also energetically unfavorable versus chain collapse. Often there are a number of nanoparticle assemblies where the polymer chains possess a similar free energy and a large barrier to rearrangement, so the different assemblies coexist in solution. In the sample shown the relative positions of the energy minima are unknown; they are shown for illustration only. Note that the polymer chain can occupy a virtually unlimited number of energetic states and the diagram is a simplified overview. TEM image adapted with permission from ref (17). Copyright 2004 American Chemical Society.

The simplest method to carry out polymer assembly is by performing a solvent switch (Figure 2a) of a preformed polymer (or rehydration of a dried thin film of polymer). For this, a block copolymer is first dissolved in a good solvent. Next, a poor cosolvent (often water) for at least one block is added to initiate assembly. Exploring this method in the 1990s gave the first reports of kinetically trapped block copolymer assemblies in solution, pioneered by Eisenberg,^14,15^ followed by Bates^16,17^ and Wooley/Pochan.^18,19^ Kinetic trapping occurs when rearrangement of chains within a polymer assembly does not occur at a sufficient rate to form the most stable product as the solvent ratio changes. This leads to nonergodicity–the formation of a nonequilibrium state where a mixture of different types of assemblies are present within polymer dispersions (Figure 2b). The assemblies in such mixtures can still evolve over time but may take months to reach their final composition.^20,21^ Careful tuning of the assembly conditions, such as adjusting salt concentration, means unusual structures, such as toroids,^18^ Y junctions,^10^ semivesicles^22^ and compound vesicles^23^ can be accessed in this way. Such morphologies cannot be accessed by phospholipid assembly. It is also possible for phospholipids to assemble into thermodynamically disfavored states (indeed, liposomes can be disfavored over flat lamella).^24^ However, the fluidity of such structures means that such assemblies tend to only exist transiently, unless an energy source (such as ATP consuming proteins^25,26^) is present to drive shape changes.

It was soon realized that such kinetic trapping of polymers can be advantageous, because it (1) permits the synthesis of hierarchical polymer assemblies that cannot be accessed under thermodynamic control^19,27^ and (2) introduces pathway dependence into polymer assembly, an intrinsic requirement for “memory” and information transfer.^28,29^ Early studies also identified the crucial role played by a plasticizer upon kinetic control.^18^ A plasticizer partially solvates the solvophobic regions of a block copolymer, increasing interchain distances and thus promoting faster chain mobility. This lowers the energy barrier to chain rearrangement, so can determine whether a system operates under kinetic or thermodynamic control.^30^

**Figure 3:**
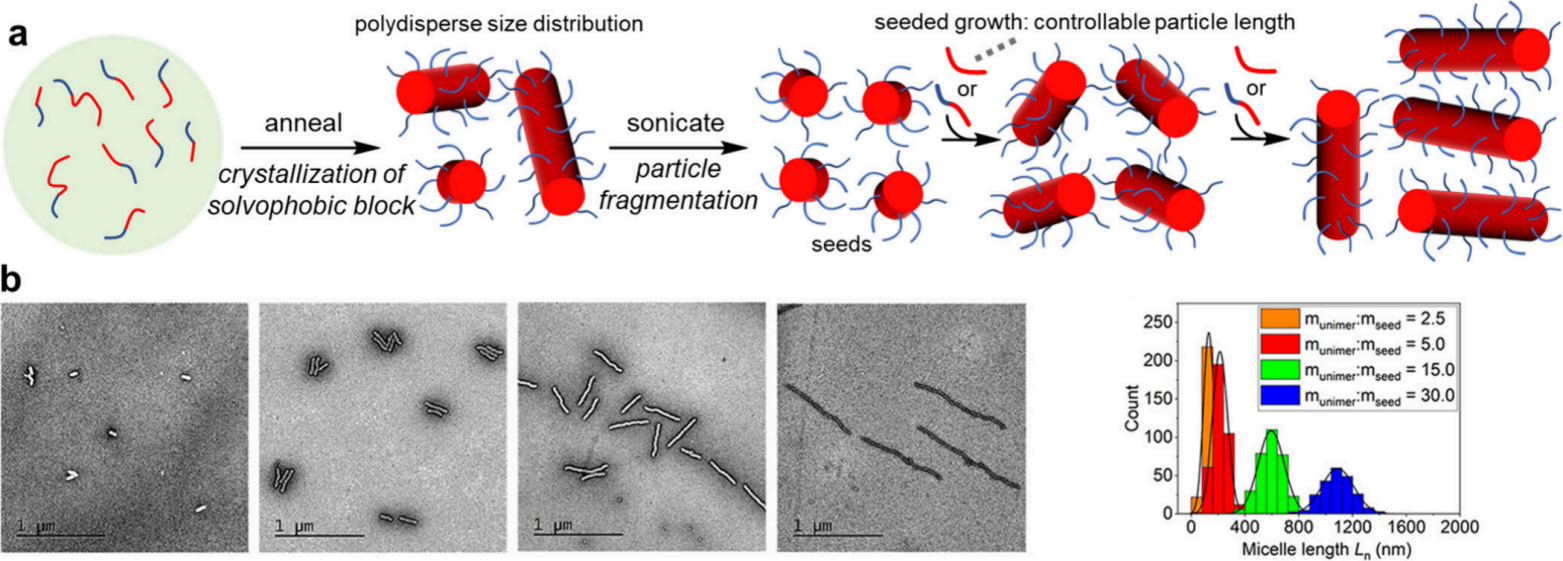
(a) Living crystallization-driven self-assembly as a sophisticated example of kinetic control over the assembly of preformed block copolymers. The polymers are first heated and then cooled to room temperature, which results in crystallization of the solvophobic block, producing cylindrical micelles of uncontrolled length. Sonication causes micelle fragmentation to produce seeds. Bidirectional epitaxial growth produces cylindrical micelles of specified length. The ability of the polymer to retain information on its thermal history is key for kinetic control over micelle growth. (b) TEM images of polymer assemblies extended by addition of unimer to seeds. Part b adapted with permission from ref (34). Copyright 2019 American Chemical Society.

Kinetically controlled assembly can be further elaborated into seeded assembly, whereby block copolymer chains containing a solvophobic region attach to a preformed assembly (a seed). Crystallization-driven self-assembly (CDSA), pioneered by Manners,^31^ is the most explored method for producing kinetically controlled polymer assemblies from seeds. In this method, an additional driving force to assembly is provided by the crystallization of suitable solvophobic polymer blocks as they are annealed. The directional nature of crystallization gives rise to the formation of structures with low curvature, such as rods and platelets. When seed particles are used to nucleate assembly “living CDSA” is possible (Figure 3a), whereby epitaxial growth from the seed continues as long as polymer is added.^32−35^ This permits the precise control of length/size and thus allows particles with multiple domains and low polydispersity to form (Figure 3b). Key to achieving this is to (1) ensure epitaxial growth is faster than self-nucleation and (2) use seeds that are near-uniform in size (achieved by sonicating a solution of polymer assemblies formed by self-nucleation).^36^

A related method reported by O′Reilly and co-workers uses complementary nucleobase pairing as a driving force to produce nonequilibrium micellar assemblies.^37^ This uses spherical seeds formed from diblock copolymers containing thymine units within a long hydrophobic chain. Addition of a diblock copolymer containing a shorter hydrophobic chain and complementary adenine units to the seeds results in 1D growth, producing worm-like micelles of controllable length. The mismatch in hydrophobic chain lengths is hypothesized to lead to steric crowding at the hydrophobic/hydrophilic interface of the particles, which is relieved by the morphological change. Only spherical micelles formed if the polymer chains are instead premixed and assembly induced by a solvent switch. This approach has recently been extended to grow nodes of controlled length on the surface of polymersomes.^38^ Combining polymers with different chemical composition is likely to prove a powerful way to fine-tune assembly shape and properties.^39^

## Kinetic Control Arising from Simultaneous Polymerization and Self-Assembly

Controlled and living polymerizations, which are used to form block copolymers, occur under kinetic control. While below the ceiling temperature polymerization is favored over depolymerization, the formation of polymer chains of narrow dispersity (i.e., a trivial form of defining sequence) is *entropically* disfavored versus the formation of highly disperse chains.^40−42^ Under thermodynamic control monomer connections would generally be random, unless an additional driving force is present to bias sequence and/or length.^43^ In controlled polymerizations, monomer self-initiation has a higher activation energy than chain extension. This provides the ability to specify the number of polymer chains (based on the amount of initiator) and the monomer sequence within a polymer. Information (i.e., sequence control) has been encoded into polymers using both chain-growth and (stepwise) step-growth polymerizations.^44^

**Figure 4:**
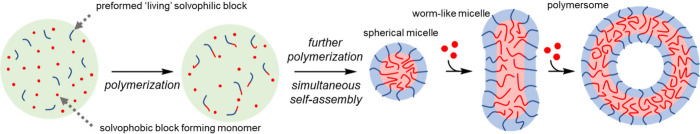
Polymerization-induced self-assembly (PISA) is a process where polymerization and self-assembly occur simultaneously. Depicted is a dispersion polymerization, whereby the solvophobic block forming monomer is initially freely soluble in solution. After a critical degree of polymerization of the solvophobic block, the polymer chains spontaneously self-assemble. After monomer is added, the entire process, including morphological evolution, happens without further intervention. Depending on the nature of the polymer and solvent, morphological evolution cannot occur at a rate comparable to that of polymerization, meaning that nonequilibrium morphologies can form transiently or persistently.

Controlled polymerizations can be harnessed to direct *in situ* polymer assembly. Rather than synthesizing a complete block copolymer in a fully compatible solvent and then subjecting it to conditions that induce self-assembly, the two processes can be made to proceed concomitantly. This is possible when a solvophilic polymer is extended with monomers that produce a solvophobic block. The resulting amphiphilic polymer spontaneously self-assembles before full consumption of the monomer forming the solvophobic block. This technique, termed polymerization-induced self-assembly (PISA), has been widely used to produce polymer assemblies in a single-step procedure (Figure 4).^45^ As the hydrophobic block lengthens, the most stable morphology typically evolves to structures with increasingly lower intrinsic curvature (as predicted by packing parameter).^46^ Usually the dominant morphology goes from spherical micelle to worm-like micelle and then to polymersome (a vesicle containing a bilayer membrane formed from polymers and a solvent filled interior compartment).

PISA is touted as an industrially relevant technique because it can be performed at high solids concentration (unlike solvent-switch procedures), uses commercially available monomers and reproducibly gives nanoparticles of narrow dispersity.^45^ However, PISA is also a powerful technique for producing nonequilibrium polymer assemblies because, depending on the properties of the formed polymer, the dynamics of self-assembly can be very much slower than polymerization.^47^ This leads to kinetic traps, where a polymer cannot assemble into its lowest energy state.^48^ As polymerization continues, the formed assemblies instead become destabilized due to an increase in strain, which can be spontaneously released by either particle fusion^49^ or result in colloidal destruction.^50^ Alternatively, formation of an additional hydrophilic block after assembly can cause the regeneration of lower order assemblies and eventual chain solubilization.^51^

It is also pertinent to note that during PISA a chemical reaction (i.e., forming and breaking of chemical bonds between monomers to generate a polymer) is directly coupled to a self-assembly process. In other words, nanoparticle self-assembly is at least in part controlled by the kinetics of the chemical reaction. Coupling self-assembly processes with networks of chemical reactions is a rapidly emerging area of interest that is currently largely focused on small molecule activation.^52−60^ Intense interest is being given to this area because it permits access to active metastable materials that display transient, adaptive or responsive behaviors, attributes that are not possible with static materials residing in a thermodynamic well.^61^ Manipulating the chemical reaction by use of different reagents, catalysts etc. directly impacts self-assembly. The use of PISA to generate such active materials derived from covalent polymers has received only limited attention; further ground-breaking studies in this area should be anticipated in the coming years.

**Figure 5:**
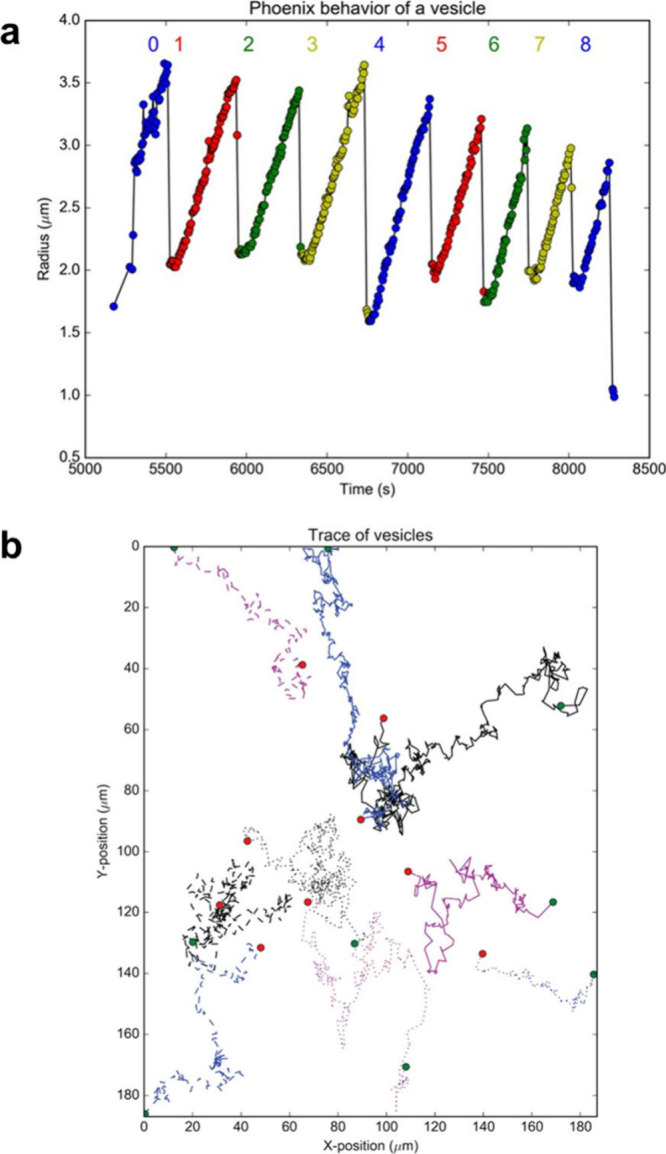
Complex behaviors observed with PISA systems. (a) Cycles of vesicle swelling and collapse are coupled with polymerization. (b) When using photoinduced electron/energy transfer PISA, the polymerization occurs faster in the center of the frame, where the light source is most bright. The resultant concentration gradient in monomer generates Marangoni flow; the movement of several vesicles toward the center is shown as traces in the position graph. Images reproduced from ref (65), licensed by a CC-BY 4.0 International License (http://creativecommons.org/licenses/by/4.0/).

Several studies from the group of Juan Pérez-Mercader have shown it is possible to combine PISA with other concurrent chemical reactions.^47^ They have shown that PISA can be initiated by radicals produced during the Belousov–Zhabotinsky (BZ) reaction^62,63^ and can be interfaced with a copper(I)-mediated click reaction.^64^ The group has also observed emergent properties arising from assemblies produced during PISA reactions. In one study, vesicles formed during PISA were observed to undergo several cycles of swelling and collapsing as the polymerization continued (Figure 5a).^65^ The authors hypothesize that chain extension of the core-forming polymer block causes the vesicles to grow larger. The structures collapse when the osmotic pressure inside the vesicle falls to a critical value (due to monomer depletion) and the membrane is weakened. Reswelling subsequently occurs as long as unreacted monomer is present. In addition to this, because the specific PISA reaction is initiated by light, the vesicles undergo Marangoni flow (Figure 5b). As the consumption of monomer molecules occurs more quickly where light is more intense, a concentration gradient along the vesicle-water interface is generated, which in turn drives particle motion.

PISA can be performed using various polymerization techniques and reaction conditions. Therefore, there is much scope for further exploration of PISA as a method for dynamic control of polymer assembly.^66^ For example, the dynamic assembly of different nanoparticle morphologies, such as worm-like micelles which entangle and form gels, should provide new ways to produce responsive and self-healing materials. This could be used to bring further utility and longevity to existing technologies, such as a conductive ionogel made using PISA.^67^

## Kinetic (Un)Trapping of Polymer Assemblies Mediated by Temperature Changes

**Figure 6:**
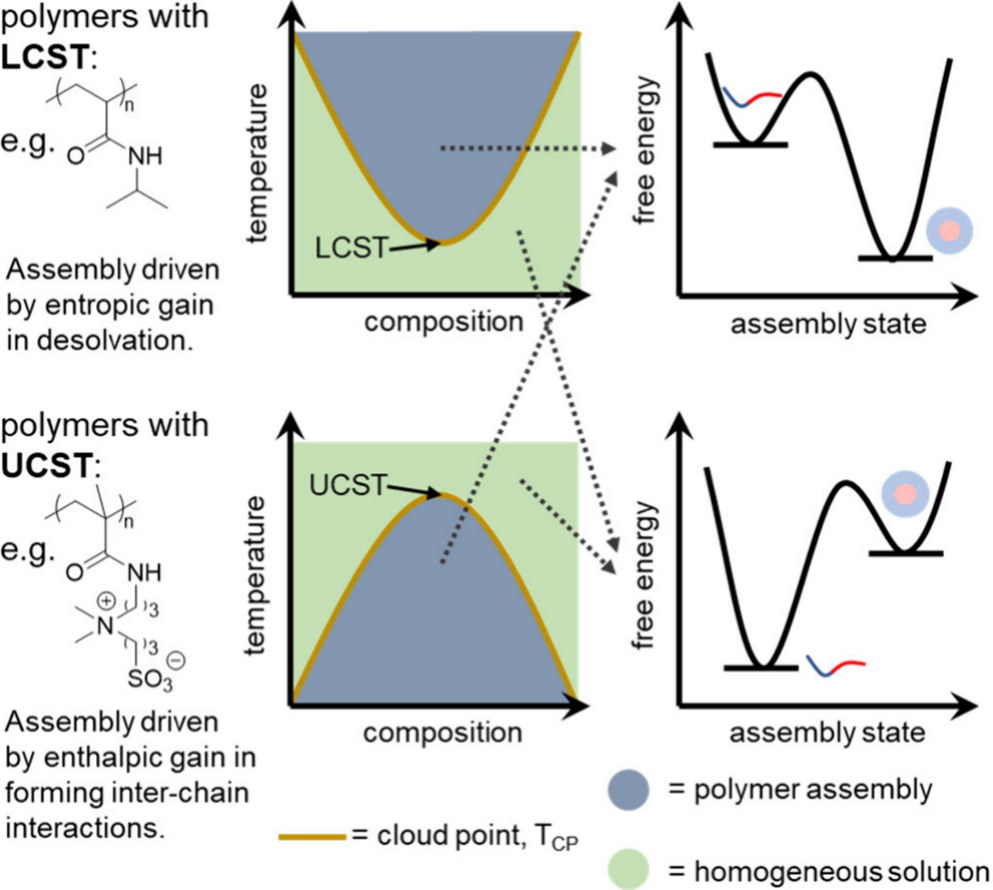
Polymers that are thermoresponsive can display either a lower critical solution temperature (LCST) or an upper critical solution temperature (UCST). The crossover temperature (cloud point, T_CP_) varies with composition (solvent ratio, polymer volume%, salt concentration, etc.).

There are many reports of copolymers containing blocks with temperature responsive solubility.^68,69^ Such a block becomes insoluble when cooled below its upper critical solution temperature (UCST, where phase separation is generally an enthalpically driven process) or heated above its lower critical solution temperature (LCST, where phase separation is generally an entropically driven process).^70^ Upon a temperature change, the responsive polymer block transitions from adopting an extended conformation to a collapsed globule. Globules then assemble together. This means that heating and cooling can be used to trigger polymer assembly and disassembly (Figure 6), which can be used to either drive a system toward or away from equilibrium.

**Figure 7:**
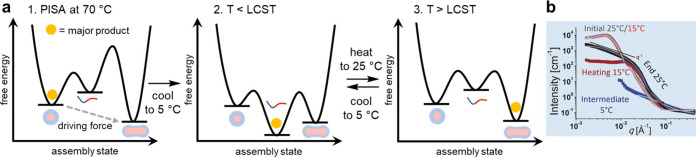
(a) Phase transitions and qualitative energy diagrams of a thermoresponsive polymer containing poly(NIPAM). The polymer is made using PISA and initially assembles into large spherical micelles. Cooling promotes disassembly, while subsequent warming allows thermodynamically favored worm-like micelles to form. Small spherical micelles (not shown) are formed as an intermediate to worm-like micelles. Toggling between worm-like micelles and disassembled polymers can be repeated multiple times. (b) Small angle neutron scattering profiles of the polymer solutions at different temperatures. Note the initial and end scattering profiles at 25 °C are not superimposable, showing different assemblies are present. Part b adapted from ref (71) with permission. Copyright 2017. John Wiley and Sons.

This was illustrated by Plamper and Warren, who studied temperature responsive copolymers formed from the *N*-isopropylacrylamide (NIPAM) monomer.^71^ Performing PISA produces large spherical micelles (Figure 7a). Lowering the temperature below the LCST for poly(NIPAM) results in complete dissolution of polymer chains and hence particle disassembly. Subsequent raising of the temperature to above the LCST promotes polymer reassembly to produce a gel of worm-like micelles–the presumed thermodynamic product. Assembly/disassembly of worms by temperature change is reversible and can be followed by neutron scattering (Figure 7b). Adjusting the assembly conditions and temperature postassembly means it is possible to force a specific polymer formulation to assemble into three different morphologies. Hence, it can be deduced that the PISA process produces kinetically trapped (nonequilibrium) spherical micelles and that the cooling/heating cycles permits relaxation toward different equilibria. Future work should explore how the free energy dissipated in such a process could be harnessed for an application, such as driving reaction networks or performing mechanical work. Mediating polymer assembly and disassembly with kinetic control in this way could prove useful for cargo release.

Plamper and co-workers also showed in a related study that it was possible to toggle between various nonequilibrium assemblies of an interpolyelectrolyte complex.

These assemblies are formed of a mixture of polymers containing oppositely charged blocks and poly(NIPAM).^72^ The polymers are first assembled in water containing plasticizing NaCl (to screen charge). Next, the assemblies are kinetically trapped by diluting the NaCl when the solution temperature is either above or below the LCST. The polymers of these two resulting solutions display different temperature responsive behaviors, assembling to give divergent structures. This is an excellent example of how “memory” can be encoded into polymer assemblies–the chemical composition of the two solutions is identical, but each behaves differently.^73,74^ Also, it is noteworthy that two “stimuli” are required^75^ to drive the system away from equilibrium: (1) “removal” of plasticizer (NaCl) and (2) adjusting the temperature to above or below the LCST. The need to coordinate multiple processes in order to generate *dynamic* nonequilibrium assembly is well understood for nonequilibrium systems, such as molecular machines.^76−78^ It is anticipated that translating this knowledge will provide many opportunities for developing smart nanotechnology.

**Figure 8:**
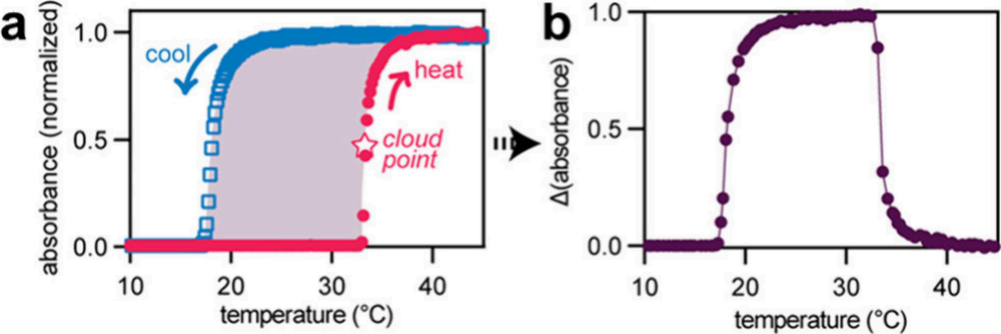
**a** Thermoresponsive polymers often display hysteresis behavior (as shown here for a polymer with a LCST). Polymer assembly, as observed by an increase in absorbance, occurs at a higher temperature than disassembly. (b) Hysteresis can be quantified by the difference in absorbance between heating and cooling at a given temperature. Figure adapted with permission from ref (79). Copyright 2023 American Chemical Society.

A recent study by Knight probed the ability of a temperature responsive polymer to display hysteresis with dynamic behavior during heating/cooling (Figure 8a).^79^ Simple polymers, formed solely from alkyl methacrylates containing an oligo(ethylene glycol) or alkyl side chain, possess a LCST and thus reversibly assemble above the cloud point temperature (T_CP_). These polymers display hysteresis behavior: T_CP_ is higher for heating than for cooling, indicating the assemblies persist at a temperature for which they are thermodynamically unstable. The magnitude of hysteresis (Δ*T*_CP_) is dependent on side chain length, hydrophobicity and temperature ramp rate (Figure 8b). It is suggested that entanglement of polymer chains hinders disassembly and produces kinetically trapped assemblies upon cooling. As the temperature ramp rate is reduced, the magnitude of hysteresis decreases, as the polymer chains have more time to disentangle. The simple structure of these polymers suggests that this simple hysteresis mechanism is likely found in many materials and thus warrants further investigation and eventual application.

Conversely, such hysteresis behavior when cooling and heating polymers with a UCST is rarer. It has been observed with ester-containing polymers in water/ethanol mixtures.^70^ Hysteresis is suggested to arise due to the complex interplay between favorable hydration of ester groups along the polymer chains and the entropic cost of stretching chains to allow this to occur during heating/cooling.

These reports show both the promise and challenges of working with thermoresponsive polymers. While it is fairly straightforward to “dial in” this behavior to a system of interest, small changes in monomer structure often can produce drastic and difficult-to-predict alterations in temperature response. For example, poly(2-substituted-2-oxazoline) in ethanol/water mixtures has a LCST when it contains an *n-*propyl side chain, while it has a UCST with an *n*-nonyl side chain.^80^ The ability to trap assemblies by “freezing” should lead to a variety of applications, such as on-demand cargo release. Intrinsically disordered proteins, naturally occurring sequence defined polymers, can also be programmed to display LCST or UCST behavior with hysteresis.^81^ Subtle changes in peptide sequences have been shown to alter phase behavior dramatically.^82^ The inherent flexibility of such proteins is key to enabling them to be responsive and dynamic. This allows them to bind multiple guests and regulate cellular pathways.^83^ Biocompatible and thermoresponsive polymers should serve as an excellent platform for mimicking these proteins.^84^

## Particle Shapeshifting without Intermediate Disassembly

There are many potential applications of polymer assemblies that undergo changes in shape and morphology without intermediate disassembly, because this preserves information on initial particle formation conditions.^85^ Examples are found in systems where unimer exchange is extremely slow.^86−90^ This is particularly useful with polymersomes,^91−93^ because cargoes are retained in the internal cavities of these assemblies. Manipulating polymersomes therefore allows temporally and spatially controlled trafficking of material.^94^ Cargo mixing occurs when polymersomes fuse together.^95^ Biology uses the fusion of vesicles formed of phospholipid membranes to traffic cargoes and regulate both intra- and intercellular processes.^96^ The same should be possible in a wholly synthetic setting with polymersome fusion, which has been achieved using various stimuli, such as photoswitching,^92^ polymerization^51^ and protein complexation.^95^

**Figure 9:**
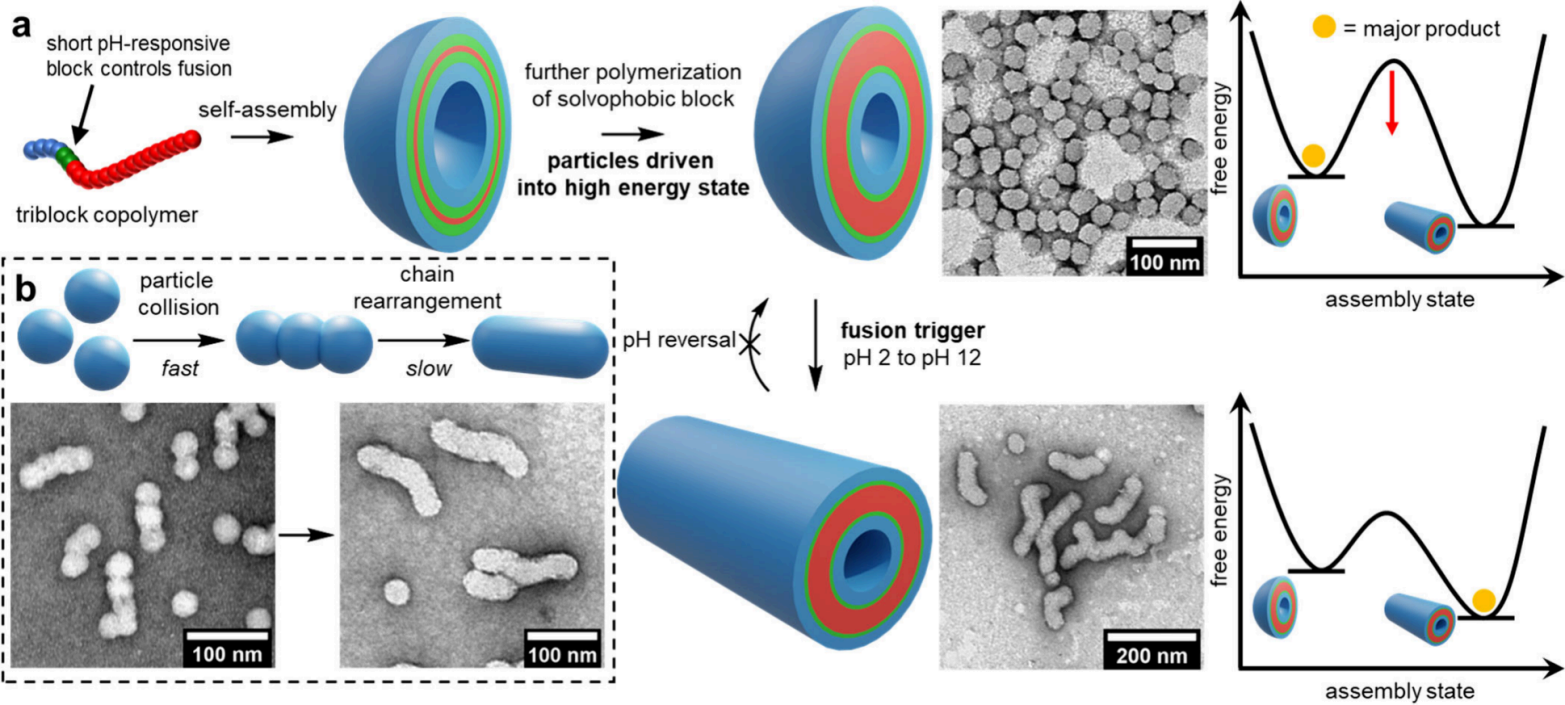
Triggered polymersome fusion. (a) Polymer nanoparticles are prepared using ROMPISA. As the polymerization of the hydrophobic block continues postassembly, the particles are driven into a high energy state as they cannot rearrange to accommodate the longer polymer chains. Fusion is initially inhibited due to the surface charge of the particles at low pH. Increasing the pH removes the barrier to fusion, resulting in the formation of tubular particles formed from multiple spherical particles. (b) The mechanism of fusion was elucidated by scattering experiments. The spherical particles rapidly collide and adhere, followed by slower chain rearrangement. (TEM image of intermediate species obtained by quenching the fusion process after five seconds). Image modified from ref (97), licensed by a CC-BY 4.0 International License (http://creativecommons.org/licenses/by/4.0/).

Recently, chemically triggered fusion of polymersomes was reported (Figure 9a).^97^ In this case, polymersomes are formed in a high energy state and then fuse once a specific chemical signal is applied. The particles are formed of triblock copolymers synthesized using ring-opening metathesis polymerization-induced self-assembly (ROMPISA). The rigid poly(norbornene) backbone means chain mobility is very slow, preventing particle rearrangement in the latter stages of polymerization. Therefore, continued polymerization “charges” the polymersomes with stress (membrane tension), the extent of which is controlled by the target polymer length. This drives the system away from thermodynamic equilibrium and thus provides a driving force for fusion. One block contains a pH responsive tertiary amine, which is protonated and hence positively charged during PISA, which is performed at pH 2. Charge repulsion between the surfaces of the formed particles prevents fusion at low pH. Once the pH is increased to deprotonate a tertiary amine, charge repulsion is removed and rapid particle fusion occurs. The fusion process is irreversible; reverting the pH back to acidic does not result in particle fission, thereby showing that the initially formed particles assemble under kinetic control. This also shows that a pH trigger lowers the activation energy to fusion, rather than perturbing the free energy of the initial and final particles. Analysis of the process by *in situ* small-angle X-ray scattering revealed a two-step mechanism, whereby deprotonated particles first rapidly adhere to each other, followed by slower chain rearrangement to minimize surface area (Figure 9b).

This work sets the stage for the development of cascades or sequences of fusion of different polymer nanoparticles. This would allow the behavior/properties of one population of nanoparticles to influence that of another, echoing interspecies biological communication.^98,99^ Controlling the crossed fusion of nanoparticles could therefore provide a method of coordinating functions such as cargo trafficking, drug delivery or sensing. Such an approach should be general to polymer nanoparticles of any fusogenic structure.

**Figure 10:**
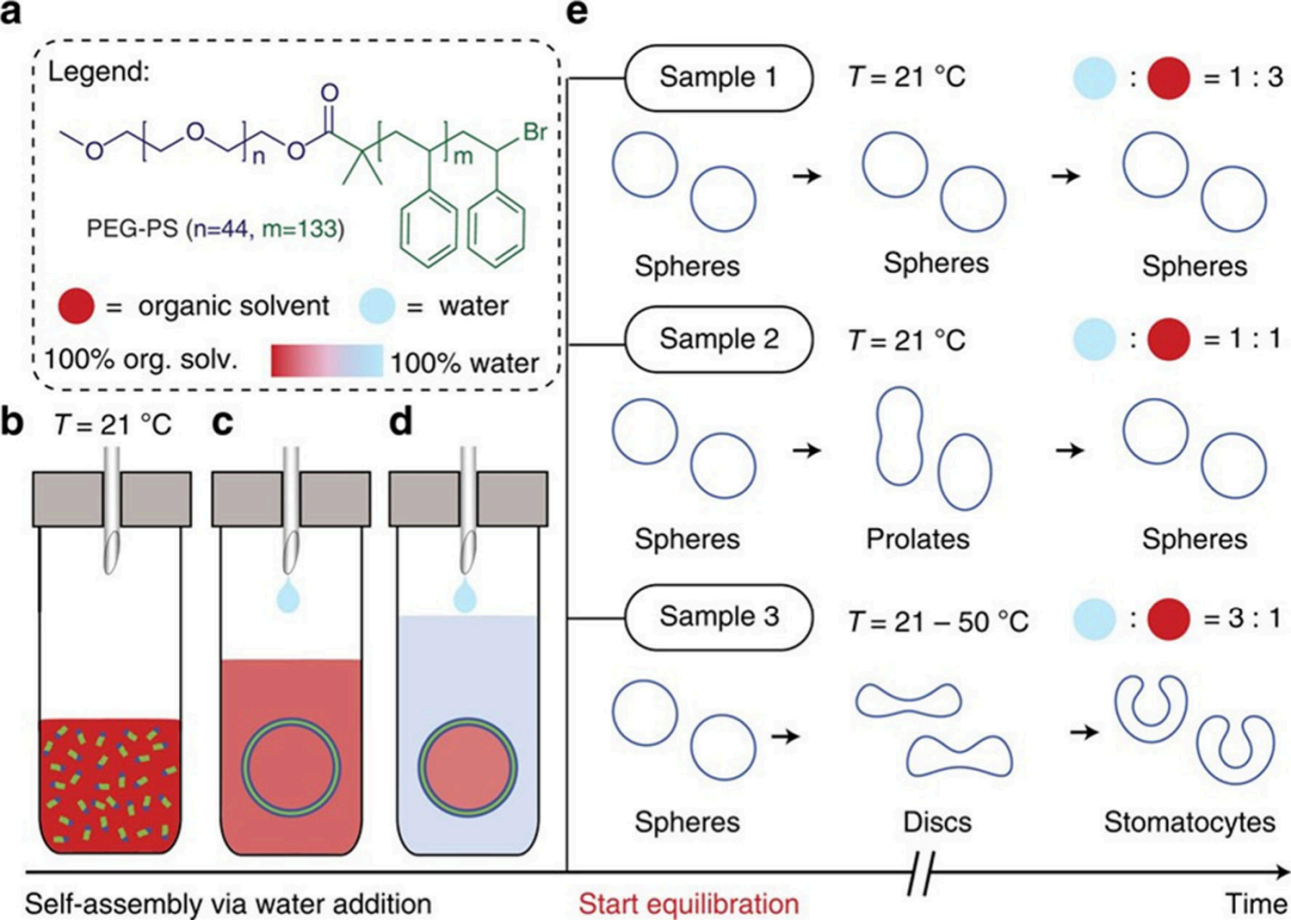
Using solvent composition to drive a system of polymer assemblies away from thermodynamic equilibrium. (a) Chemical structure of diblock copolymer. (b) The polymer is first dissolved in an organic solvent mixture (THF/dioxane). (c) Upon self-assembly, the solvent composition is identical inside and outside the polymersomes. (d) Continued addition of water reduces the permeability of the polymersome membranes and generates osmotic imbalance—the polymersomes are now in a high energy state. (e) The polymersomes relax to equilibrium by deforming in different ways depending on the solvent composition. Image reproduced from ref (103), licensed by a CC-BY 4.0 International License (http://creativecommons.org/licenses/by/4.0/).

In addition to fusion, polymersomes can be manipulated postassembly to adopt a variety of morphologies, some of which constitute nonequilibrium states.^100,101^ The selective permeability of a polymersome membrane can be exploited to force a shape transformation. Osmotic pressure is introduced by varying the ratio of good to bad solvent,^102,103^ changing salt concentration,^104^ or by the addition of a solute unable to permeate across a membrane, such as PEG.^105−107^ This drives a shape transformation of the polymersome, which persists until pressure equilibration. It was also found that the solvent composition can be used to adjust the time taken for polymersomes to equilibrate to a thermodynamically favored structure after nonequilibrium assembly (Figure 10).^103^

**Figure 11:**
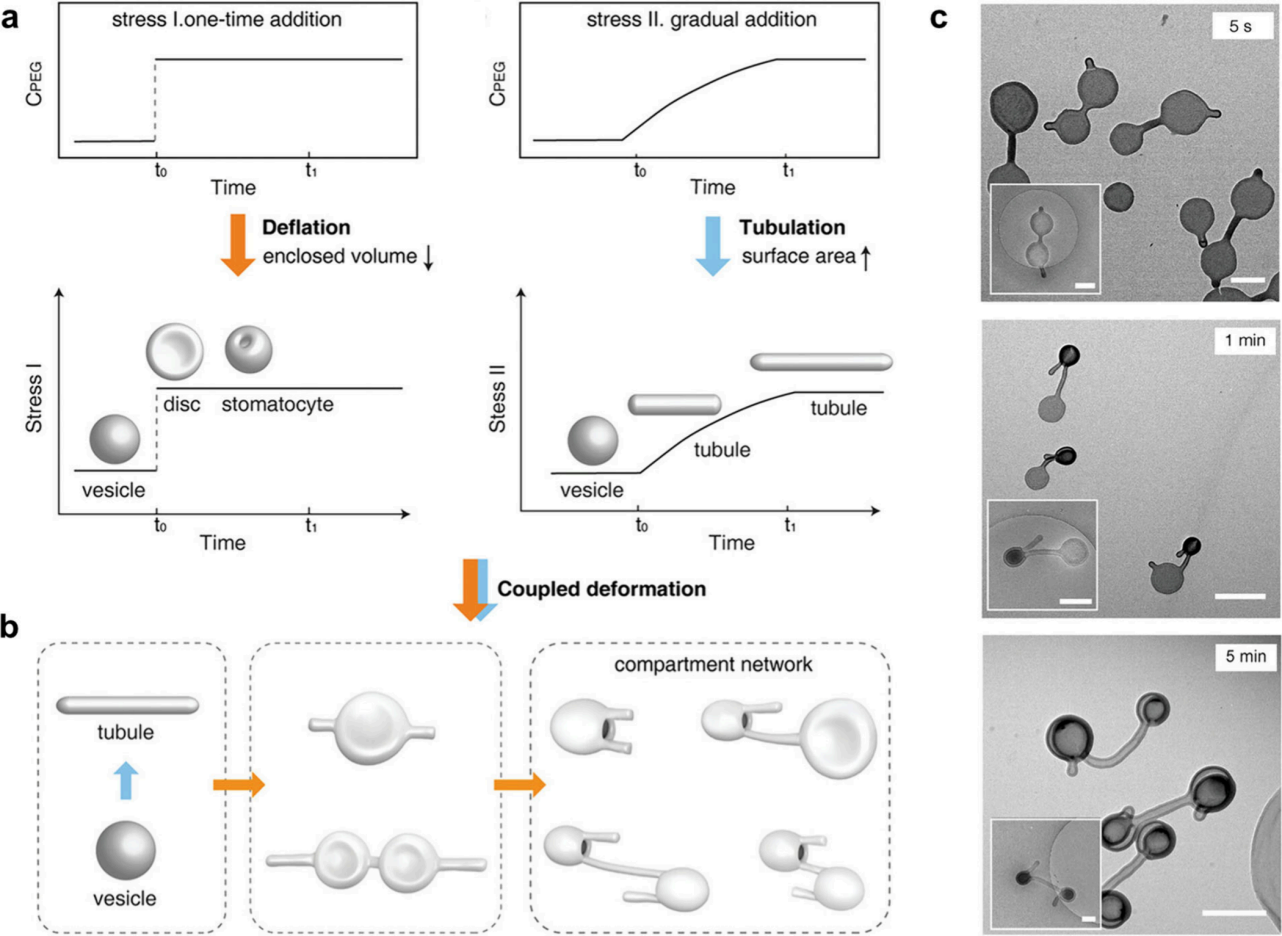
Using osmotic shock to construct connected networks of polymersomes. (a) Polymersomes can be either deflated or elongated by increasing the concentration of PEG quickly or slowly. (b) By coupling these two methods of polymersome deformation, networks of polymersome compartments linked by tubes form. (c) TEM images of the resultant networked compartments, which evolve in shape over time. Scale bar = 1 μm. Image modified from ref (108) licensed by a CC-BY 4.0 International License (http://creativecommons.org/licenses/by/4.0/).

The process of perturbing preformed polymersomes was further elaborated by Wilson and co-workers (Figure 11).^108^ They found that stress could be imparted onto the initial spherical polymersomes in two ways, generating different particle shapes: (1) by a step change in osmotic pressure due to the addition of PEG, causing stomatocyte formation due to particle deflation or (2) by instead gradually adding PEG to induce tubule formation. The different response to the two stimuli implies that the system is pushed into a nonequilibrium state by the addition of PEG. By combining these two “stimuli”, it is possible to form complex particle shapes, where compartments are linked together by tubules. This is an excellent example of how the rigid nature of polymers is advantageous for controlling pathway dependent assembly. It should be noted that the polymersome lumen extends across entire particles, meaning contents can freely traverse between compartments without needing to pass through a membrane. This seminal study opens many new directions of research because it shows that simple polymers can controllably deform into complex structures. Hopefully it will be possible to use this system as a platform for applications which may, for example, take advantage of positioning catalytic units into different compartments within the same particle.

## Transient Perturbation of Polymer Assemblies by Integrating Chemical Reaction Networks

Recent years have seen the emergence of a new field – Systems Chemistry – that aims to explore the emergent properties that arise from networks of interacting molecules and other synthetic systems.^109^ At the current time most Systems Chemists have interests in supramolecular polymerization^110^ and small molecule activation.^111^ Kinetically controlled assembly results when these processes are interfaced with chemical reaction networks. This allows a variety of properties, such as viscosity,^112^ molecular motion^76^ and chirality expression^56^ to be controlled and regulated over time. For example, supramolecular polymerization can lead to exponential^113^ or oscillating^55^ growth of fibers. Generally speaking such systems return to their original state once the chemical reaction is complete if assembly is thermodynamically disfavored. However, kinetic trapping of disfavored assemblies can also occur.^114^

The study of covalent polymers within this field is burgeoning. Several of the systems discussed above make use of networked chemical reactions or environmental changes to manipulate polymer assembly. Here the use of chemical reaction networks to transiently perturb polymer assemblies will be discussed in more detail. The versatility and utility of covalent polymers mean they offer a wealth of functional nanotechnology to integrate within reaction networks. A typical example of this is the integration of the BZ reaction with polymers to affect oscillating micellization and vesicle formation.^115,116^

**Figure 12:**
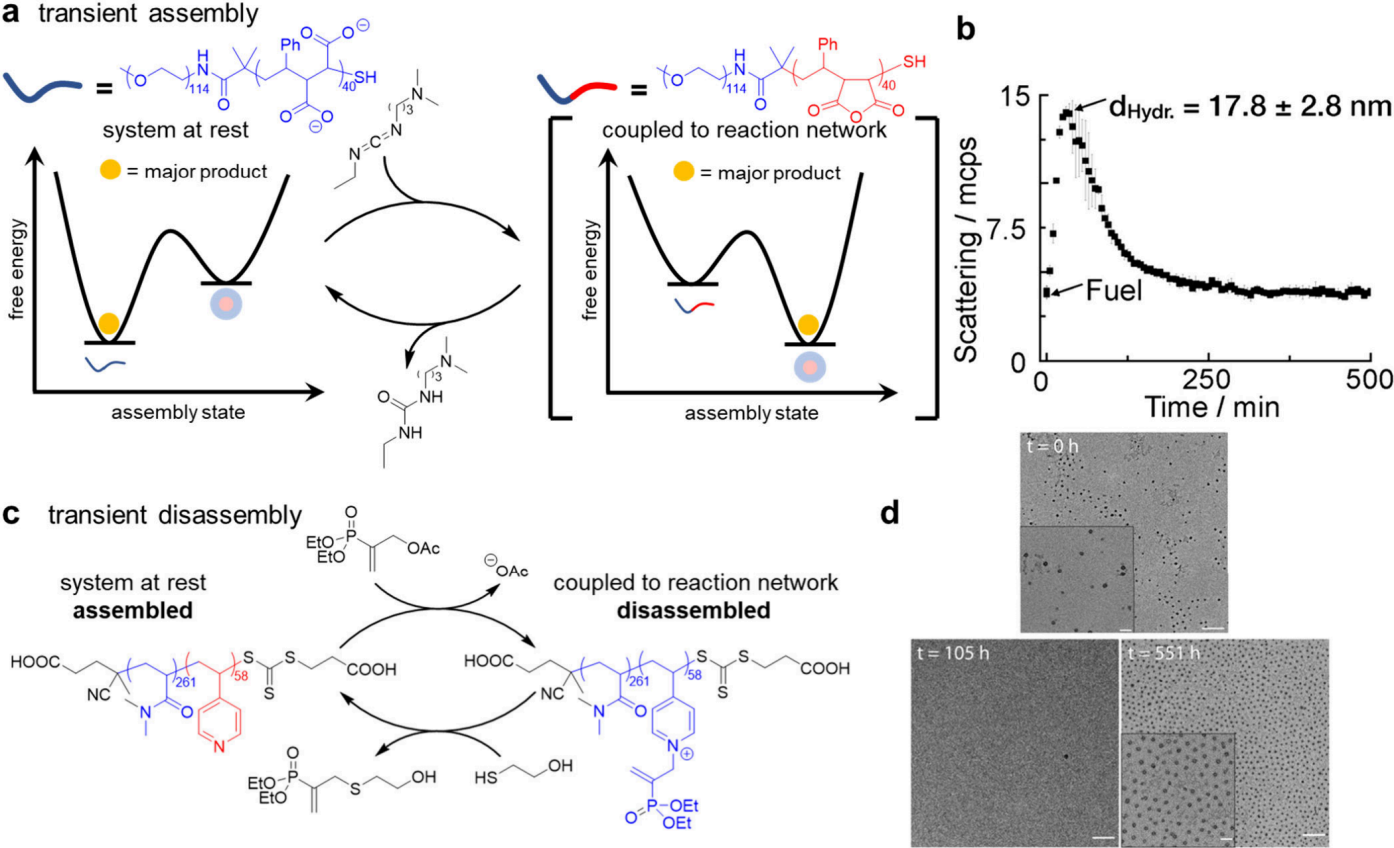
Transient assembly and disassembly of polymers controlled by chemical reaction networks. In both cases the polymer catalyzes the degradation of a high energy molecule. (a) Dehydration of carboxylates to produce anhydrides using EDC produces amphiphilic polymers, which transiently self-assemble until the anhydrides are hydrolyzed. (b) Transient assembly is evidenced by a short-lived increase in scattering intensity. (c) Alkylation of a hydrophobic pyridine side chain using diethyl(α-acetoxymethyl) vinylphosphonate generates a positively charged pyridinium, rendering the entire polymer water-soluble and driving disassembly. Nucleophilic attack by a thiol removes the vinylphosphonate and triggers reassembly. (d) TEM images taken during the course of transient disassembly. At *t* = 0 h and *t* = 551 h micelles are present, while no micelles are observed at *t* = 105 h. Scale bar = 200 nm for main image and 50 nm for insert. Section b reproduced from ref (117); section d modified from ref (119), both references licensed by a CC-BY 4.0 International License (http://creativecommons.org/licenses/by/4.0/).

Arguably the reason why most studies involving reaction networks focus on small molecules is because they can be more precisely characterized by techniques such as nuclear magnetic resonance than polymers. Therefore, a fertile area of research would be the direct translation of such studied systems to covalent polymers. Rather than being a incremental advance, this represents a great opportunity to enhance the properties (e.g., thermoresponsive behavior) of covalent polymers with emerging methods of kinetic control and nonequilibrium state generation. A seminal example of this approach was reported by Boekhoven, Walther and co-workers, who showed that anhydride formation by a reaction network could be used to drive transient micelle assembly (Figure 12a,b).^117^ This system had previously been optimized with small molecules.^118^ A carbodiimide reagent (providing a driving force for dehydration) generates a hydrophobic anhydride group from two hydrophilic carboxylate units found within the block of a copolymer. This renders the block hydrophobic and leads to polymer assembly. However, the anhydride is unstable and slowly hydrolyzes to regenerate carboxylates. This causes the polymer chains to resolubilize and results in polymer disassembly. This means that micelles are formed for a programmable amount of time; they are *kinetically sustained* and only persist as long as the carbodiimide is present to regenerate anhydride. These micelles also act as temporary nanoreactors/catalysts by colocalizing reagents to accelerate a prototypical Diels–Alder reaction. One can imagine that even greater control over the polymer assemblies could be achieved by addition of a thermoresponsive polymer block; a greater number of kinetically sustained/trapped states would result.

A similar approach, achieving the opposite effect (transient disassembly) was shown by Eelkema and co-workers,^119^ who used temporary amine side-chain quaternization to control cargo release (Figure 12c,d). In both cases, the polymer is integrated into a reaction network by catalyzing the degradation of a high energy substrate. In addition, there have been several reports detailing the use of enzymes encapsulated in polymersomes to effect feedback-controlled temporary pH fluctuations by degrading an added reagent to produce acid or base.^120−123^

Chemical reaction networks have also been integrated with polymers to control active coacervates, droplets that form upon liquid–liquid phase separation when macromolecules associate in solution. Often such phase separation is driven by charge attraction between different macromolecules, so charge neutralization can be leveraged to control coacervation. Spruijt and co-workers demonstrated coacervate growth by phosphorylation of adenosine diphosphate (i.e., an increase in charge attraction).^124^ Boekhoven and co-workers used their aforementioned transient anhydride formation to produce temporary coacervates containing RNA.^125^ Eelkema has further elaborated on these approaches by achieving transient coacervation using fully synthetic polymers^126^ and within gels.^127^

**Figure 13:**
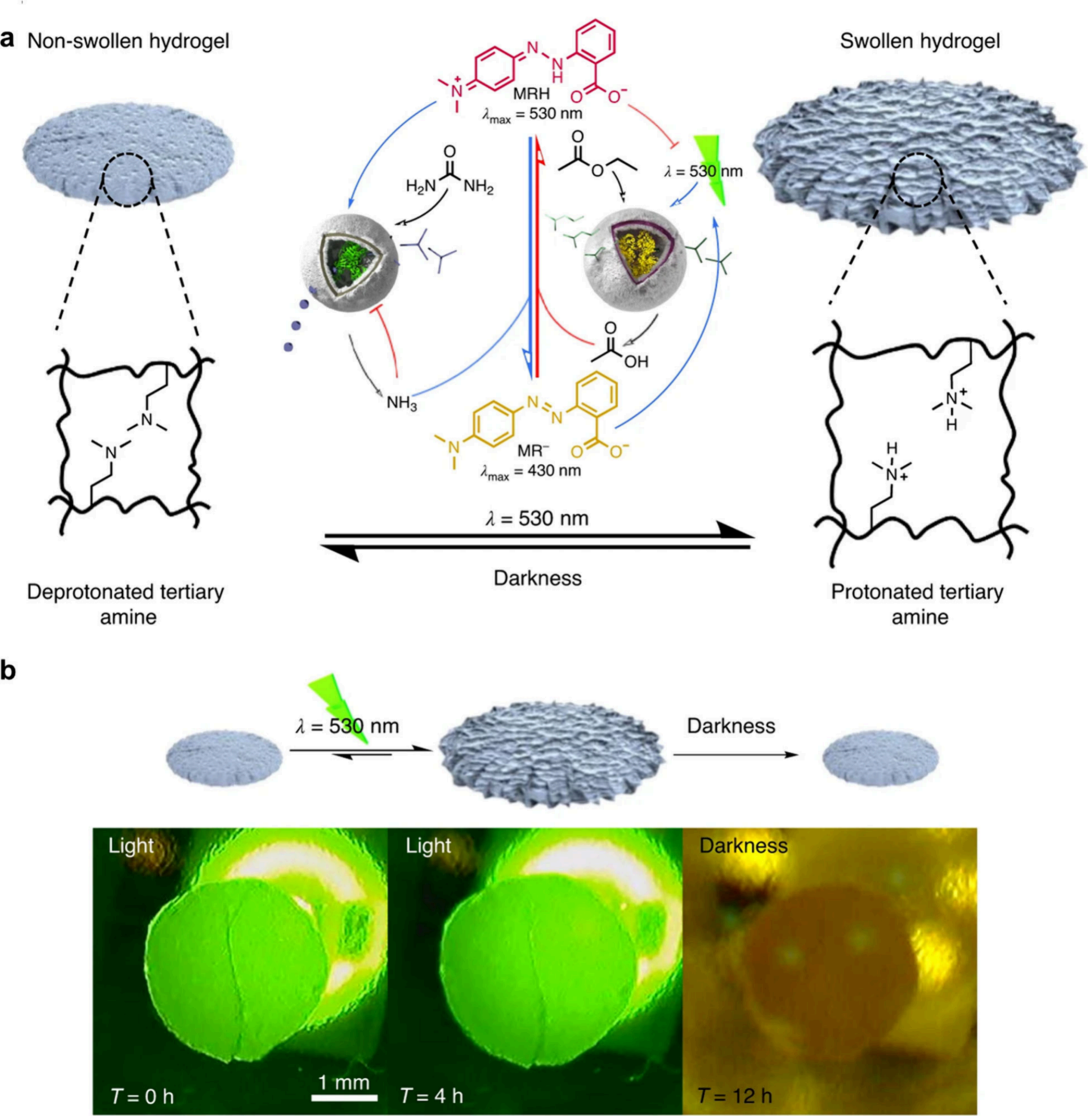
Feedback loops by interfacing two populations of polymersomes. (a) One polymersome population contains an acid generating esterase and has a membrane whose permeability can be reversibly increased by light irradiation. The other population contains a base generating urease, which counteracts the activity of the esterase. The pH reaches two different steady states in darkness (high pH) and under light irradiation (low pH). (b) The polymersome network was incorporated into a hydrogel containing pH sensitive tertiary amine units. The gel swells under light irradiation and shrinks once the light source is removed. Figure adapted from ref (128), licensed by a CC-BY 4.0 International License (http://creativecommons.org/licenses/by/4.0/).

Another recent example, from Rifaie-Graham, Stevens and co-workers, highlights the potential of interfacing multiple polymersome species with chemical reaction networks^128^ and how this could ultimately be used to model cell-like behavior.^129^ In this study, two polymersome populations are used, each encapsulating a different enzyme (Figure 13). One polymersome contains a covalently bound donor–acceptor Stenhouse adduct (DASA) within the membrane. This acts as a photoswitch for permeability; exposure to green light results in DASA isomerization, which permits substrates to diffuse into the polymersome lumen. Within the lumen is an esterase, whose activity lowers solution pH by catalyzing the hydrolysis of an ester. Decreasing pH has two effects: (1) it increases the absorbance of a small molecule dye in bulk solution, which screens the DASA polymersomes from the light source, producing a negative feedback loop and (2) it increases the activity of a urease, encapsulated in an unresponsive polymersome formed by PISA. This enzyme catalyzes the degradation of urea to ammonia, which acts to increase solution pH. The combination of these processes produces oscillatory type behavior when the light source is added and removed. Embedding the polymersomes into a pH responsive hydrogel allows swelling behavior to be transiently increased by application of the light source (Figure 13b). This study represents a new level of sophistication in generating systems-

level behavior with polymer assemblies, with a single stimulus (light) controlling several chemical reactions and hence communication between polymersome populations. The temporal evolution of kinetically trapped polymer assemblies is a promising method of integrating synthetic and biological systems in order to control rheology and drug delivery.

Finally, another approach to achieve transient assembly is to use kinetically sustained cross-linking. A chemically driven example by Konkolewicz, Hartley and co-workers used a carbodiimide reagent.^130^ Several studies have looked at how the photo Diels–Alder reaction between triazolinediones and naphthalenes can be used to generate transient cross-links.^131,132^ In the dark this reaction is thermodynamically disfavored with respect to the retro reaction. Irradiation of a solution containing a bis-triazolinedione linker and a polymer possessing naphthalene side chains produced a gel network. The storage modulus of this gel was shown to increase with irradiation time until photocuring was complete. In darkness, the gel weakened continuously until it fully degraded back to a solution. Hence, the formed network only persists as long as an energy source is present.

As well as looking to small molecule research, there are examples of polymer modification that could be repurposed to develop systems that display transient behaviors. For example, Houck, De Geest and co-workers recently reported a mechanism to functionalize PEG, a ubiquitous, cheap and biocompatible polymer, with triazolinedione side groups under light irradiation.^133^ The resulting hemiaminal ether is hydrolytically unstable and breakage of the polymer chain was observed in aqueous solution. If both triazolinedione attachment and hydrolysis could occur in the same conditions, this methodology may prove an innovative method to transiently functionalize and then disassemble PEG.

## Discussion and Future Perspectives

Kinetic control over assembly is responsible for the rich diversity and behavior displayed by block copolymers in solution. From the first studies several decades ago, it was evident that polymer assembly in solution could produce diverse structures that were thermodynamically unstable but kinetically trapped. As controlled chain-growth polymerizations have become used routinely over the past 20 years, it is now simple to construct polymer assemblies with exotic shapes and targeted dimensions. New techniques such as CDSA and PISA exploit kinetic control to produce nonequilibrium polymer assemblies. Development of stimuli-responsive polymers have led to studies of dynamic rearrangement of assemblies, which can include phenomena such as assembly hysteresis and polymersome fusion. Finally, the integration of polymer assemblies within reaction networks has given rise to self-regulating and feedback behavior.

How can this research be leveraged by other fields? As Schrödinger famously noted in his book, *What Is Life? The Physical Aspect of the Living Cell*, published 80 years ago (based on lectures he gave at Trinity College, Dublin), living matter ‘evades the decay to equilibrium’.^134^ When inanimate matter is placed in a uniform environment, it dissipates energy until it ‘fades away into a dead, inert lump...’, We must be cautious in associating kinetic control with making a system “animate”; as Schrödinger explains, delaying the fall to inevitable equilibrium is not sufficient: ‘These ultimate slow approaches to equilibrium could never be mistaken for life...’ While our understanding of the natural world has greatly evolved since Schrödinger proposed these ideas, these simple facts of “living” and “nonliving” remain unchallenged. Therefore, using *static* kinetic trapping to form nonergodic systems, as possible by various methods of polymer assembly, is not enough to produce synthetic matter with active characteristics. However, polymer chemists are beginning to develop methods of *dynamic* or *sustained* kinetic control over assembly; these may play a key role in producing materials that are active, can adapt and behave somewhat autonomously. Polymers can be designed to contain an unlimited combination of chemical functionality. Crucially, such molecular design can also be used to mediate nanoscale assembly–the concurrent ordering across length scales is another hallmark of living material.^135^

A recent essay^136^ on Schrödinger’s book by Rob Phillips provides inspiration for developing matter that displays animate properties. Phillips suggests the hydrogen atom is a “quintessential test case” to understand many fundamental concepts in physics, such as the behavior of atoms, nuclei and the interactions between radiation and matter. In other words, the simplicity (and abundance) of hydrogen atoms allowed these phenomena to be explored in their least complicated manifestations. Hydrogen has therefore played a vital role in the development and understanding of physical principles, as it is one of the few analytically solvable two-body systems in physics.^137^ Phillips also argues, ‘The study of living matter needs its hydrogen atoms.’ That is to say, discovering simple systems that display behaviors or properties similar to life will provide test cases that give scientists the opportunity to fully understand the chemistry that underpins biological matter. A bottom-up approach, which starts with nonliving matter and builds complexity step-by-step, may be a suitable method for discovering rudimentary systems that have “living characteristics”.^138^ The versatility and controllability of polymer chemistry permits stepwise increases in chemical complexity to be designed. Simply making a polymer from a larger set of monomers, or controlling dispersity, allows the complexity of a system to be selected.

Three essential processes found in living matter are compartmentalization, metabolism and replication, which are all manifestations of kinetic control.^139^ Could these processes one day be produced using covalent polymers? Achieving this would surely have great impact in the areas of polymer recycling and degradability. Clearly, compartmentalization is readily achieved with polymer assemblies, most notably by encapsulation within polymersomes. But what about the other processes? Metabolism requires a system to use an energy source to synthesize its constituent building blocks and connect them together. As discussed above, chemical reaction networks are beginning to be interfaced with polymers (and importantly polymerization - PISA). Pérez-Mercader has already shown that PISA systems can show animate type properties,^140^ where the consumption of monomer can be regulated and thus be regarded as a primitive synthetic equivalent to metabolism. This has allowed resource competition,^141^ growth/death cycling^65^ and feedback^64^ of polymer assemblies to be observed.

Future studies should look toward dynamic (de)polymerization, whereby monomers can be both consumed and released by a covalent polymer chain. This would allow complete recycling of material and, as long as an energy source is present, one can expect such systems to show adaptive and responsive behavior. This would complement results found with dynamic supramolecular polymerization.^142^ Research should also focus on how to build on these seminal studies and allow us to learn how to design polymer assemblies capable of synthesizing building materials (monomers?) and catalyzing their incorporation.

Replication requires information transfer from a parent structure to its offspring. Given that Biology uses macromolecules to store (DNA) and transfer (RNA) information, achieving replication using synthetic macromolecules should be possible.^143^ Initial studies have shown that the properties of a polymer (molecular weight, dispersity) can be translated from one polymer to another by templated polymerization.^144,145^ Further research is required to produce exponential self-replication and autocatalysis of polymers.^146^

In conclusion, covalent polymers provide the ideal platform for developing assemblies that are subject to kinetic control. The structural and functional diversity of synthetic polymers, as well as the ability to control their properties across length scales makes them ideal for advancing nanotechnology over the coming years. Accordingly, further research to merge Systems Chemistry with Polymer Nanotechnology will permit the construction of synthetic systems capable of displaying emergent behaviors, which may perhaps one day be seen as ‘Biology’s Hydrogen Atoms’. As polymer chemists are already well integrated with industry and healthcare, further research in kinetically controlled assembly of polymers is a promising way to bridge the gap between fundamental blue-skies research and real-world applications.

## Abbreviations

CDSA: crystallization-driven self-assembly
LCST: lower critical solution temperature
NIPAM: *N*-isopropylacrylamide
PEG: poly(ethylene glycol)
PISA: polymerization-induced self-assembly
TEM: transmission electron microscopy
UCST: upper critical solution temperature
